# Supplementary figures and images for: Formation of a giant unilocular vacuole via macropinocytosis-like process confers anoikis resistance
Source: eLife. 2024 Nov 7;13:RP96178. doi: 10.7554/eLife.96178 (PMC11542918; doi:10.7554/eLife.96178)

Figure3 (D)

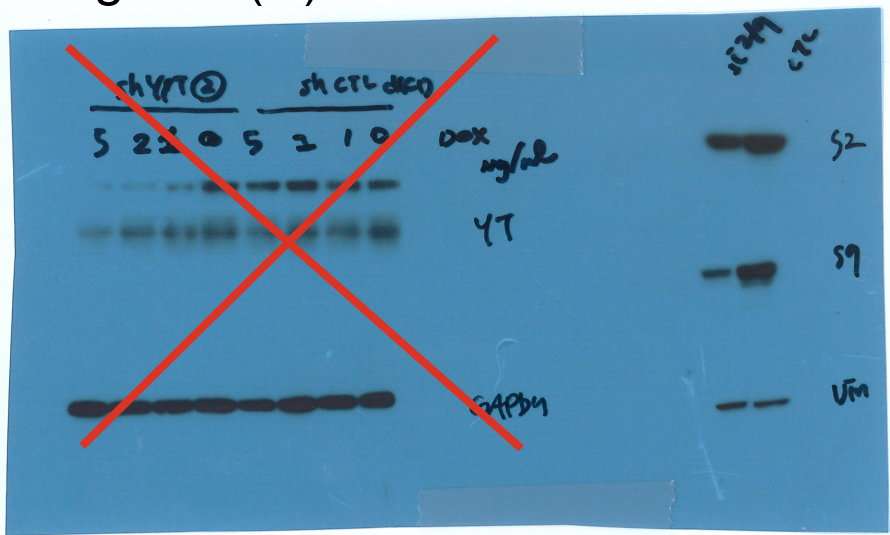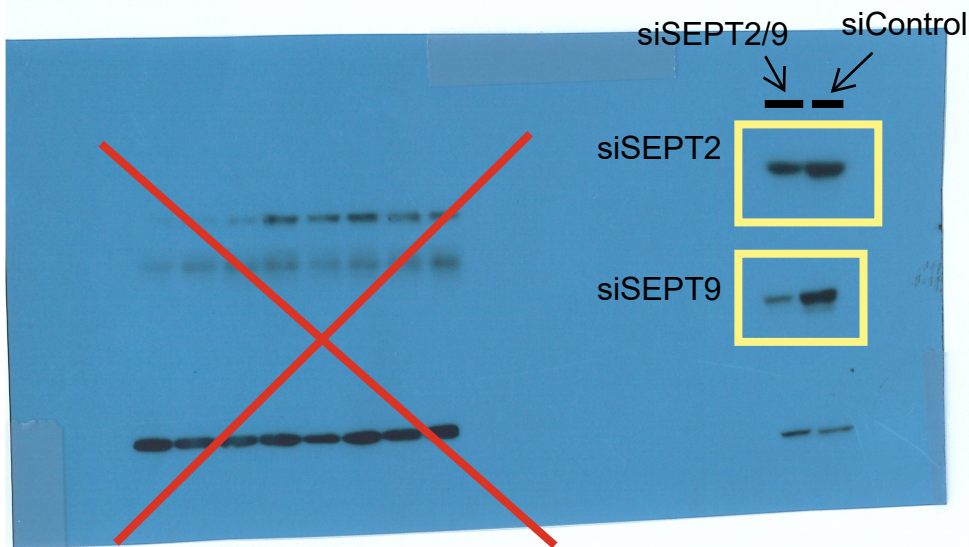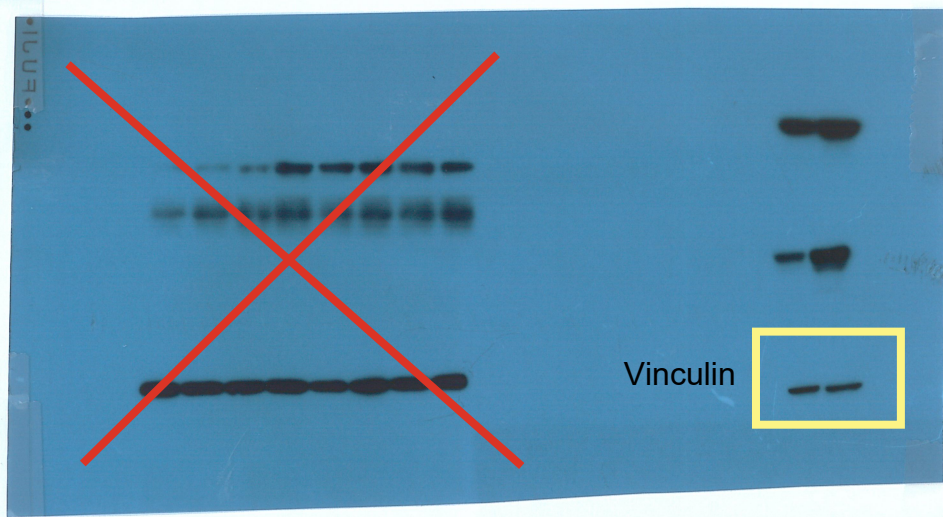

Supplement: Figure 3—source data 7. [file elife-96178-fig3-data7.zip › Figure 3-source data 7.pdf]

Figure3 (F)

230418

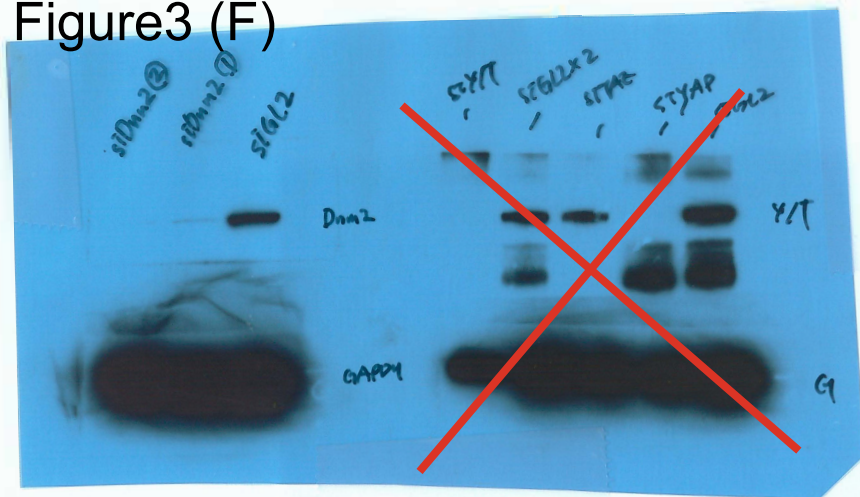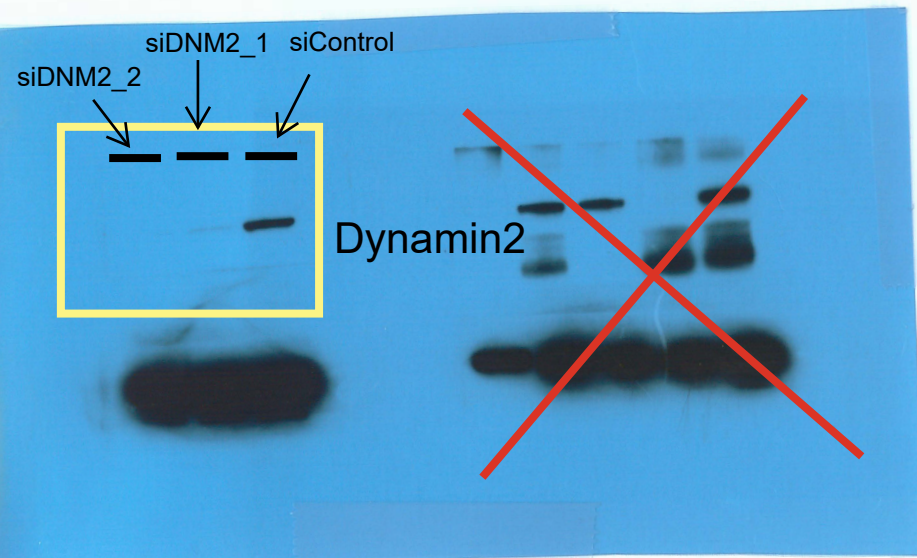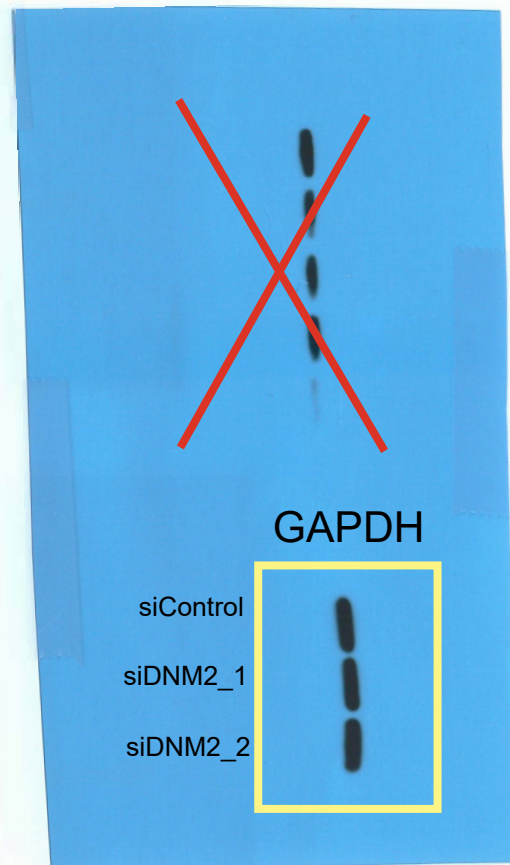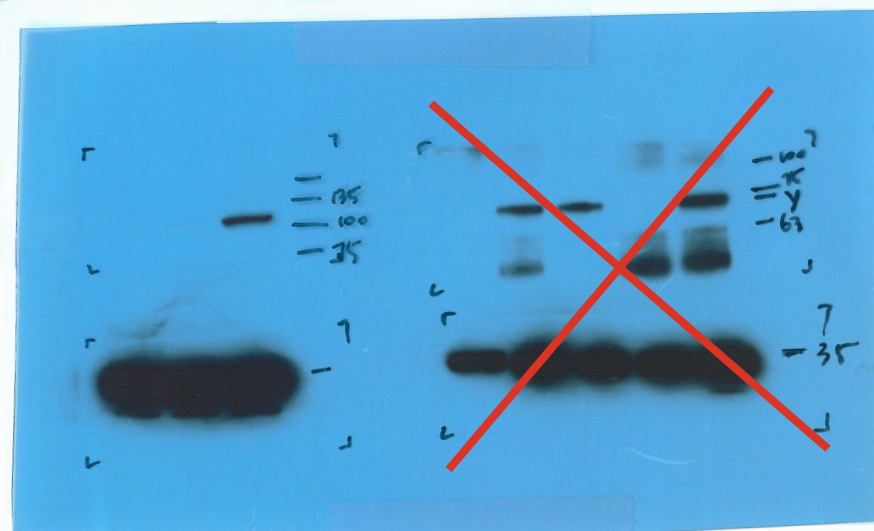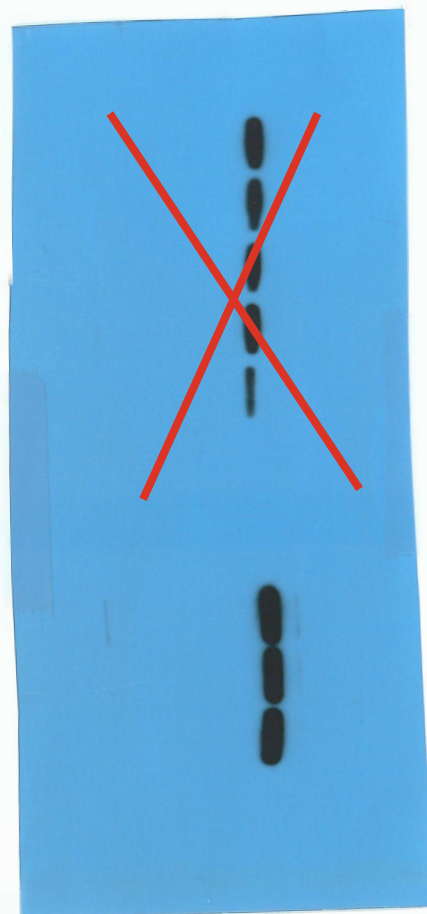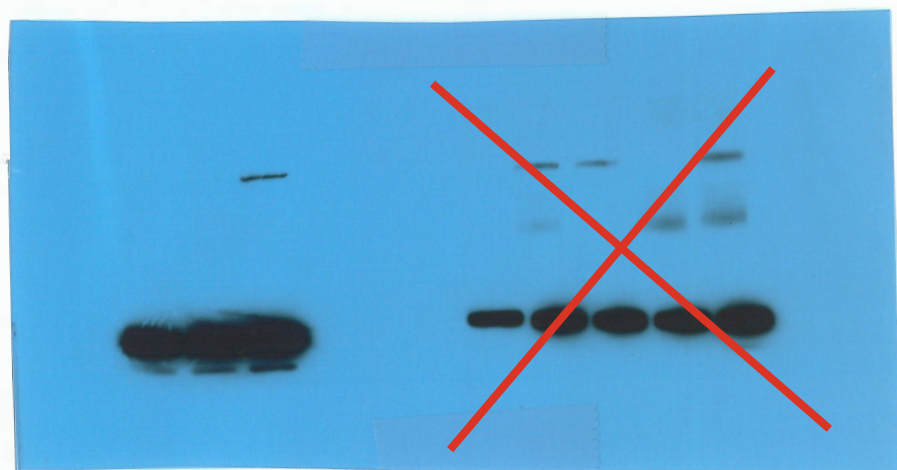

Supplement: Figure 3—source data 9. [file elife-96178-fig3-data9.zip › Figure 3-source data 9.pdf]

# Figure 4-figure supplement 1 (C)

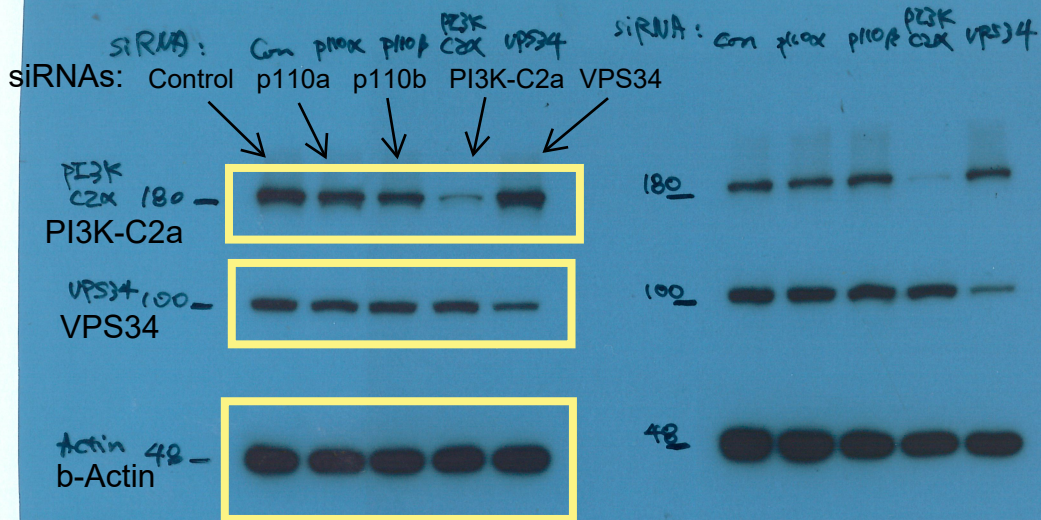

Supplement: Figure 4—figure supplement 1—source data 1. [file elife-96178-fig4-figsupp1-data1.zip › Figure 4-figure supplement 1-source 1.pdf]

21.07.29

Figure 4-figure supplement 1 (D)

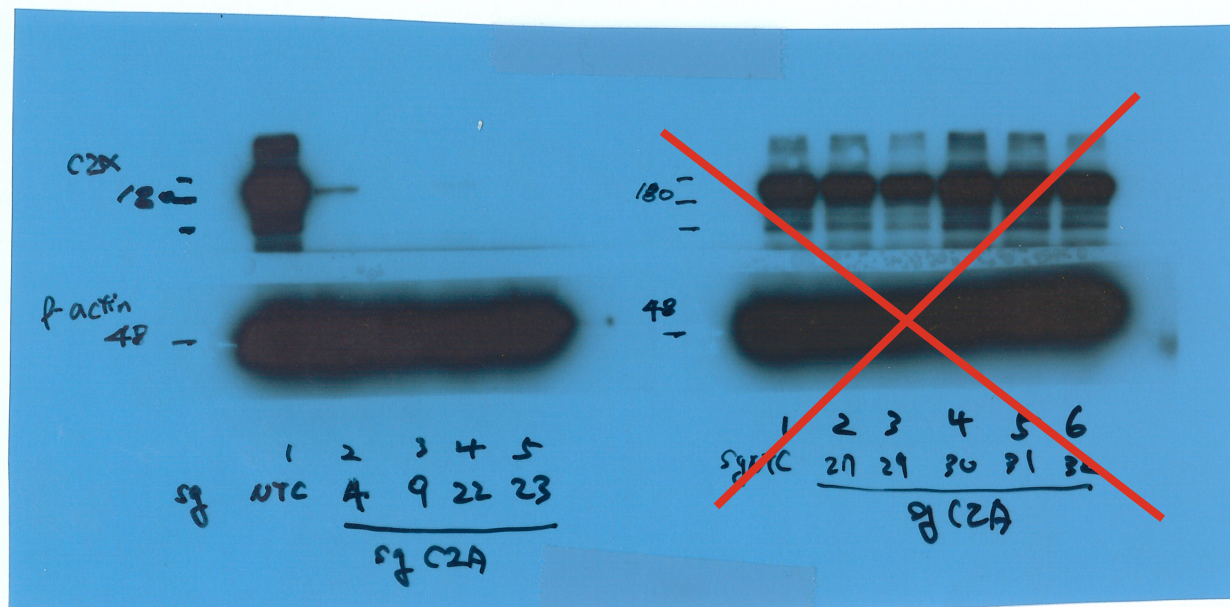

sgNTC sgPI3K-C2a

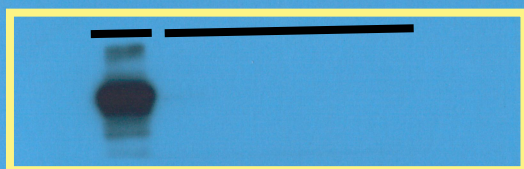

PI3K-C2a

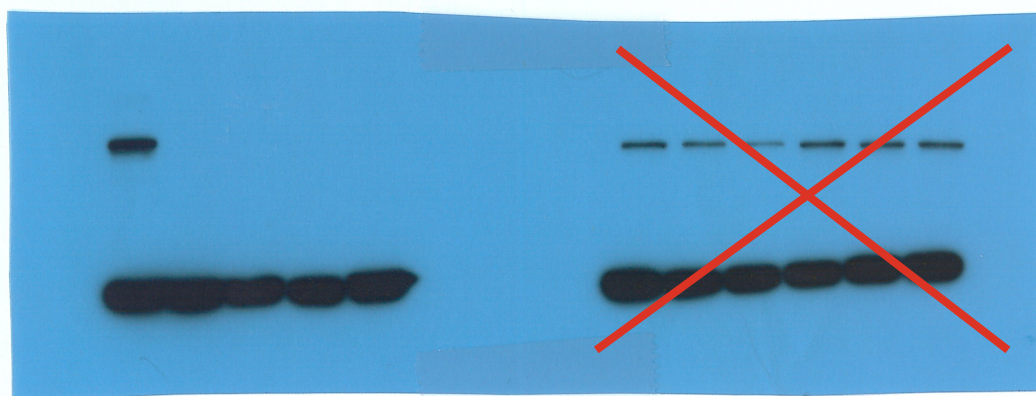

b-Actin

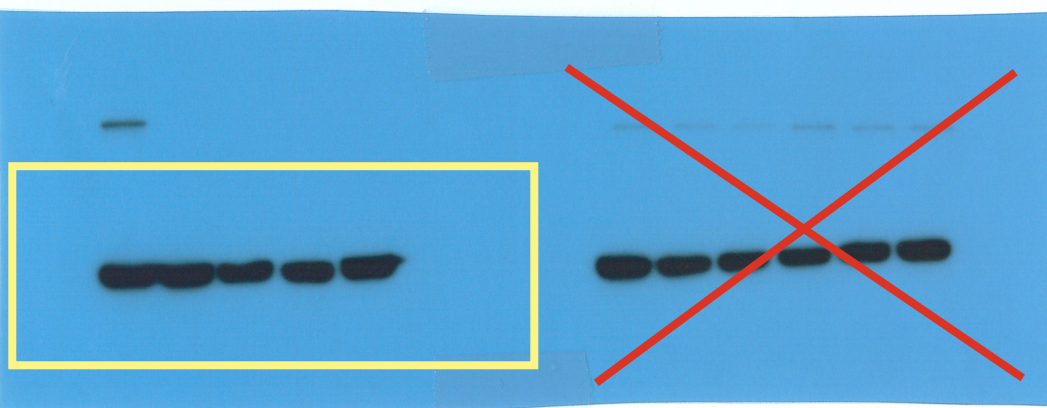

Supplement: Figure 4—figure supplement 1—source data 3. [file elife-96178-fig4-figsupp1-data3.zip › Figure 4-figure supplement 1-source 7.pdf]

# Figure 4-figure supplement 1 (E)

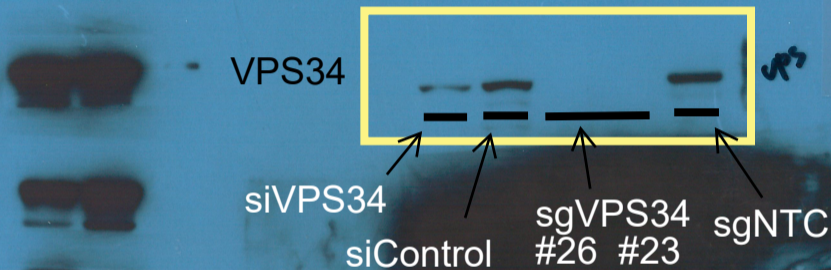

Figure 4-figure supplement 1 (E)

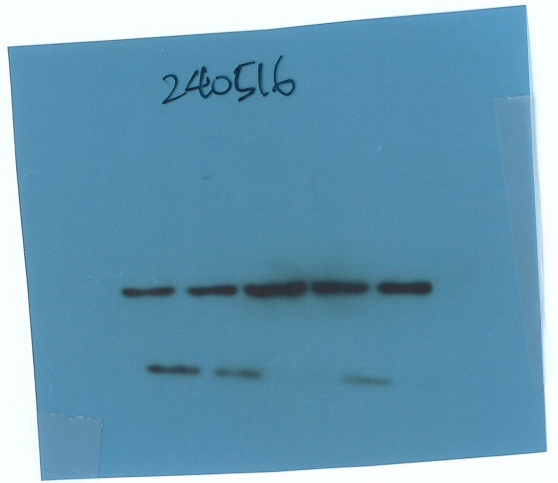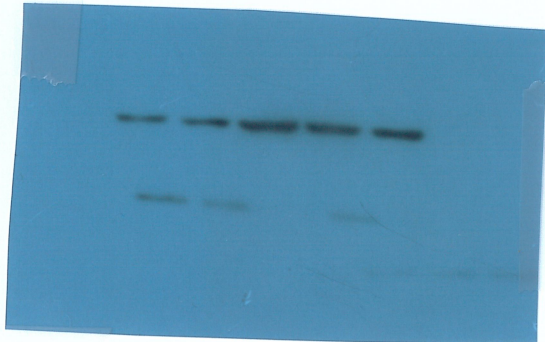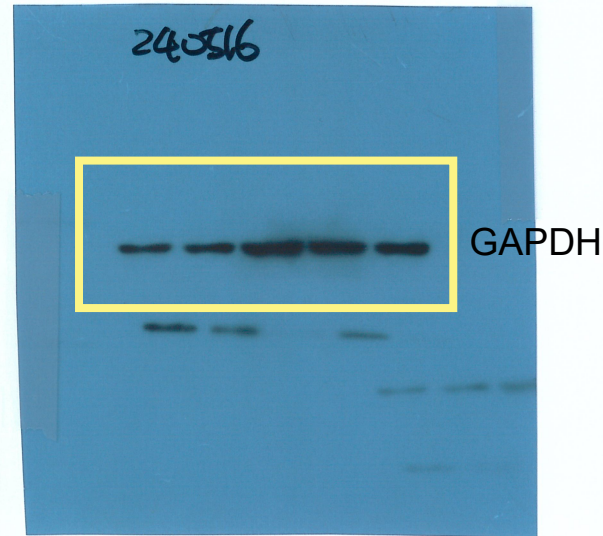

Supplement: Figure 4—figure supplement 1—source data 5. [file elife-96178-fig4-figsupp1-data5.zip › Figure 4-figure supplement 1-source 9.pdf]

1/28 Sample Figure 5-figure supplement 1

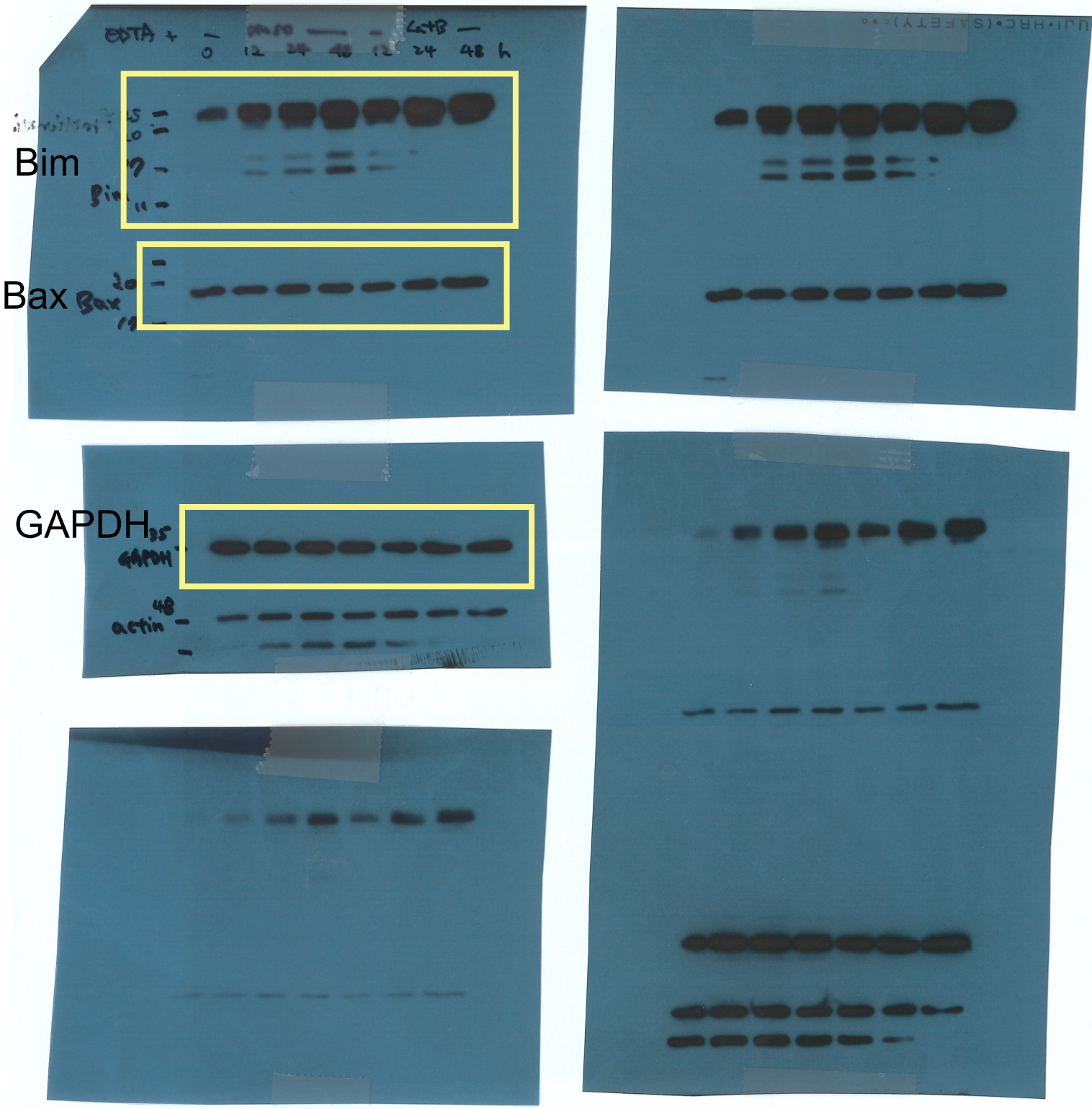

## Figure 5-figure supplement 1

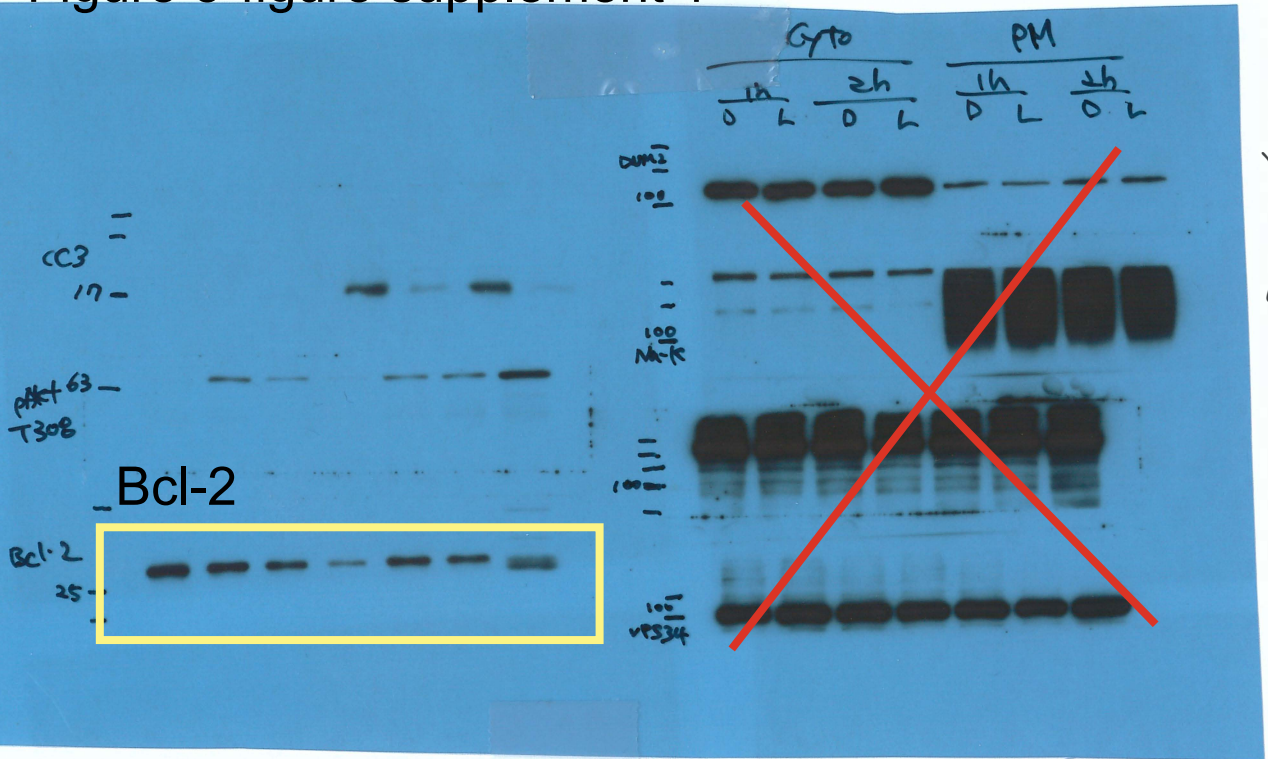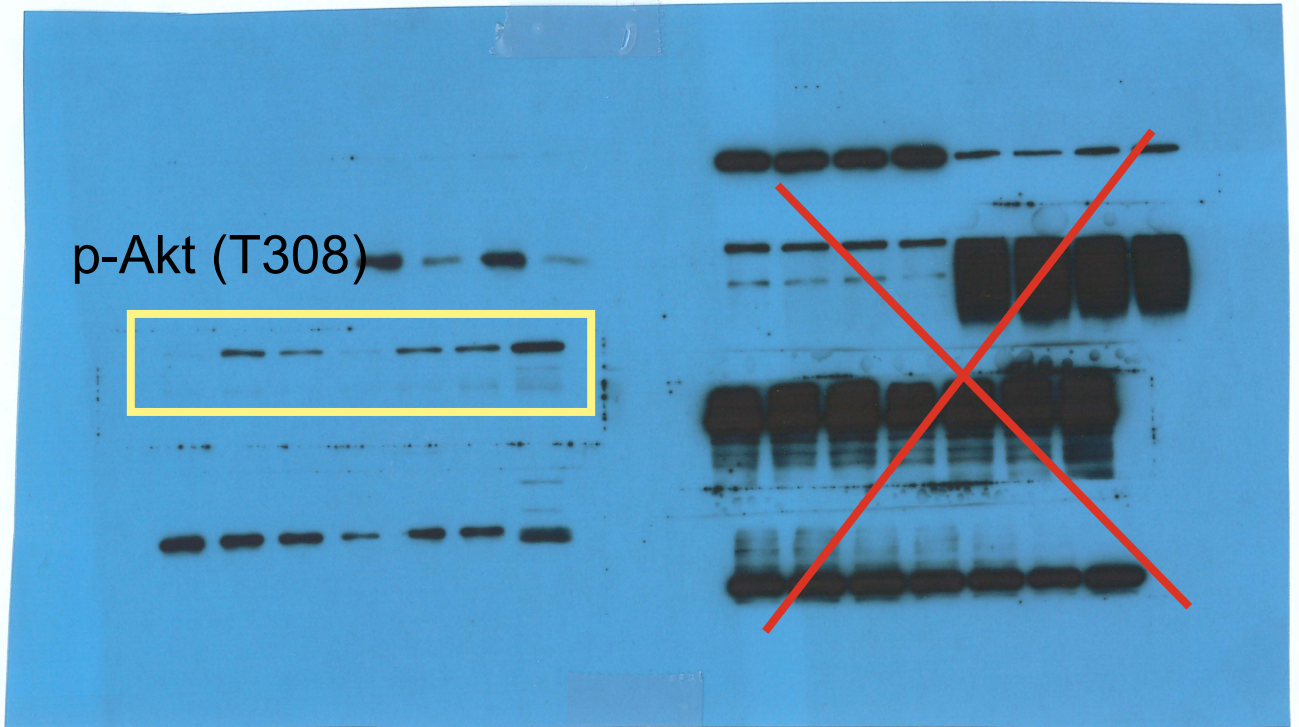

# Figure 5-figure supplement 1

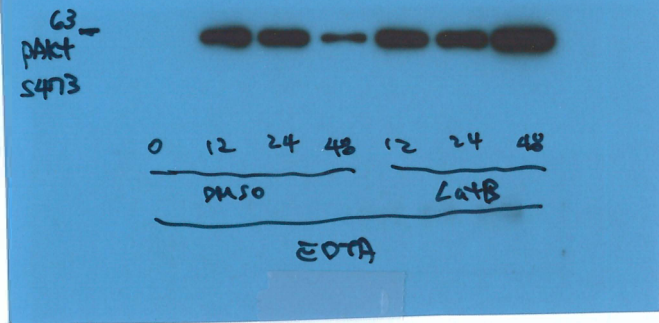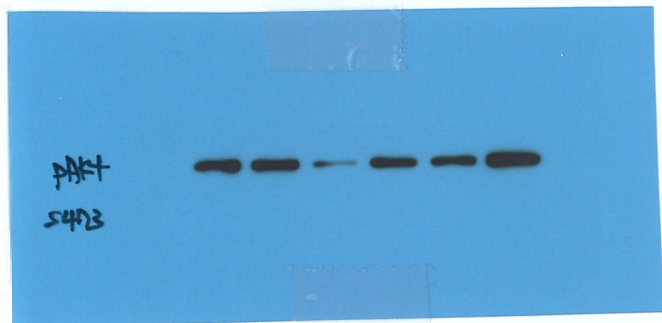

p-Akt (S473)

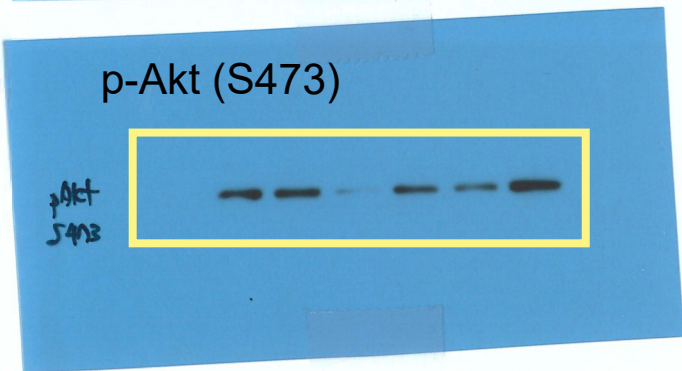

Supplement: Figure 5—figure supplement 1—source data 1. [file elife-96178-fig5-figsupp1-data1.zip › Figure 5-figure supplement 1-source data 1.pdf]
